# Analyzing Multiple Social Determinants of Health Using Different Clustering Methods

**DOI:** 10.3390/ijerph21020145

**Published:** 2024-01-28

**Authors:** Li Zhang, Olivio J. Clay, Seung-Yup Lee, Carrie R. Howell

**Affiliations:** 1 Department of Biostatistics, University of Alabama at Birmingham, Birmingham, AL 35233, USA; 2 Department of Psychology, University of Alabama at Birmingham, Birmingham, AL 35233, USA; oclay@uab.edu; 3 Department of Health Services Administration, University of Alabama at Birmingham, Birmingham, AL 35233, USA; slee9@uab.edu; 4 Department of Medicine, Division of Preventive Medicine, University of Alabama at Birmingham, Birmingham, AL 35233, USA

**Keywords:** SDoH, exploratory factor analysis, hierarchical clustering, latent class analysis, PRAPARE

## Abstract

Social determinants of health (SDoH) have become an increasingly important area to acknowledge and address in healthcare; however, dealing with these measures in outcomes research can be challenging due to the inherent collinearity of these factors. Here we discuss our experience utilizing three statistical methods—exploratory factor analysis (FA), hierarchical clustering, and latent class analysis (LCA)—to analyze data collected using an electronic medical record social risk screener called Protocol for Responding to and Assessing Patient Assets, Risks, and Experience (PRAPARE). The PRAPARE tool is a standardized instrument designed to collect patient-reported data on SDoH factors, such as income, education, housing, and access to care. A total of 2380 patients had complete PRAPARE and neighborhood-level data for analysis. We identified a total of three composite SDoH clusters using FA, along with four clusters identified through hierarchical clustering, and four latent classes of patients using LCA. Our results highlight how different approaches can be used to handle SDoH, as well as how to select a method based on the intended outcome of the researcher. Additionally, our study shows the usefulness of employing multiple statistical methods to analyze complex SDoH gathered using social risk screeners such as the PRAPARE tool.

## 1. Introduction

Social determinants of health (SDoH) [1] impact a person’s physical and mental health and wellbeing, and include social and economic factors such as housing, employment, and education, as well as area-level factors such as neighborhood SES, access to healthy food sources, and healthcare availability [2]. These factors contribute to health disparities [3,4], where certain populations experience worse health outcomes and higher rates of chronic conditions. In response, healthcare providers have begun to recognize the importance of addressing SDoH [5] to improve health outcomes and promote health equity, including the need to screen for social risks at the medical encounter.

The utilization of measures related to social and environmental determinants of health within the EMR to guide population health initiatives has gained significant traction in recent years [6] with an emergence of standardized tools designed to capture SDoH information accurately and comprehensively in the EMR. One such tool is the Protocol for Responding to and Assessing Patients’ Assets, Risks, and Experiences (PRAPARE) [7], developed to assist healthcare providers in identifying and addressing SDoH among patient populations through the electronic medical record (EMR) platform. The PRAPARE collects self-reported information from patients during healthcare encounters on a range of SDoH factors, including housing, food security, transportation, and employment. The PRAPARE also collects address information of residence for geocoding, to link to public area-level data. The tool has become a resource for healthcare providers to better understand the needs of their patients and develop targeted interventions to address SDoH challenges. For health providers and health services researchers, PRAPARE is a valuable tool for designing and customizing healthcare services to meet the specific needs of diverse patient populations. Using information from PRAPARE, providers can make informed decisions on resource allocation, program development, and policy formulation to improve the effectiveness of health services and enhance health equity in communities.

However, analyzing the data collected with PRAPARE can be challenging due to the complexity and multidimensional nature of SDoH [8]. PRAPARE surveys collect a wealth of data encompassing various SDoH domains, leading to large and complex datasets. Many SDoH variables and observations are interconnected, making it crucial to recognize patterns and structures within the data.

Despite the widespread use of PRAPARE, study is limited regarding which SDoH measures matter most [8], and which groupings of SDoH measures have the greatest impact on specific chronic disease outcomes [9] or social risk of specific group of patients [10]. Utilizing advanced statistical methods that group SDoH may help extract meaningful insights from the data and identify patterns and relationships among the variables.

Here, we describe three statistical approaches we recently used to analyze PRAPARE data for a pilot study that sought to identify and link social risk phenotypes to diabetes and obesity status among patients using PRAPARE data. These approaches include exploratory factor analysis, hierarchical clustering analysis, and latent class analysis. We also present the lessons learned as we compared the methods and eventually selected one methodological approach in presenting our pilot clinical findings.

Exploratory factor analysis [11,12,13] is a technique used to reduce a large number of variables into a smaller set of factors by identifying relationships and commonalities among multiple observed variables. This strategic reduction not only simplifies the screening process, but also enhances the efficiency and interpretability of the subsequent analytical results. For instance, in the context of PRAPARE data, it might reveal that several variables related to income, education, and housing quality can be collectively understood as an “Economic Stability” factor. Observed variables associated with anxiety, depression, and stress may load onto a common factor called “Mental Health.” Researchers can then use a set of questions to represent specific domains or factors, thus honing in on key areas to focus interventions.

Hierarchical clustering analysis [14,15,16,17] groups data points or patients based on observed similarities or differences in their variable values. It results in sets of clusters, where data points within each cluster are more similar to each other than to those in other clusters. For instance, it can identify a cluster of patients with similar experiences of housing instability, food insecurity, and transportation barriers.

Latent class analysis (LCA) [18,19] is also used to identify groups of individuals based on their responses to multiple categorical variables. However, it goes beyond clustering observed variables; its objective is to identify unobserved (latent) classes within a population based on patterns in the observed categorical responses. For instance, it can reveal distinct subgroups of individuals with specific combinations of socio-economic factors that influence their health outcomes.

Overall, using these statistical techniques to analyze PRAPARE data can help healthcare providers and researchers better understand the complex relationships between social determinants of health and health outcomes, and develop more effective strategies for addressing health disparities and inequities.

## 2. Materials and Methods

### 2.1. Study Design

This study aimed to explore the use of three statistical techniques—exploratory factor analysis, hierarchical clustering, and latent class analysis—to analyze data collected using the PRAPARE (Protocol for Responding to and Assessing Patients’ Assets, Risks, and Experiences) tool from a sample of 2380 patients who completed the PRAPARE survey in a primary care setting at our institution. The PRAPARE survey consists of questions that assess SDoH in five domains: (1) demographic information, (2) economic stability, (3) neighborhood and physical environment, (4) health care access and utilization, and (5) social and community context [20]. We used 16 self-reported variables from the PRAPARE—fear of a partner, veteran status, housing stability, incarceration history, housing insecurity, stress levels, social support, resource needs, safety in the current living environment, educational attainment, employment status, transportation access, insurance coverage, migrant or refugee status, and language proficiency. We also used 3 measures of area-level SDoH by census-tract of participant’s residence at time of assessment for these analyses: the Social Vulnerability Index overall score, rural urban commuting code, and the Yost SES Index [21]. Demographic variables such as age, sex, and gender were used to describe our results but were not included in factor analysis or latent class analysis [9].

### 2.2. Statistical Analysis

The SDoH variables were first recoded to ensure that all variables started from 1, and to facilitate interpretation of the results, such that higher values were indicative of higher risk. Descriptive statistics were then employed to summarize the frequencies and percentages for categorical variables, providing insight into the distribution of the data.

We conducted exploratory factor analysis, hierarchical clustering, and latent class analysis (LCA). All statistical analyses were carried out using the R software package (version 4.0.5). Exploratory factor analysis was conducted using the ‘fa’ function from the R package ‘psych’ (version 2.2.5). Hierarchical clustering was performed using the ‘NbClust’ function in R (version 3.0.1), and latent class analysis (LCA) was conducted using ‘poLCA’ (version 1.6.0).

#### 2.2.1. Exploratory Factor Analysis

Factor analysis is a statistical technique that is used to reduce data to a smaller set of summary variables, and to explore the underlying theoretical structure of the phenomena. Here, we used exploratory factor analysis (EFA) [22] as a technique to identify the underlying factors that contribute to the observed variation in the data. The number of constructs or factors was determined based on the number of eigenvalues greater than 1 [23]. To evaluate and verify the constructs found in the exploratory factor analysis, we used confirmatory factor analysis (CFA). The evaluation criteria included root mean squared error of approximation (RMSEA), standardized root mean residual (SRMR), and goodness of fit index (GFI) [24,25]. RMSEA measures the discrepancy between the model and the observed data, with lower values indicating a better fit. SRMR measures the difference between the model-implied and observed covariance matrices, with values less than 0.08 indicating a good fit.

#### 2.2.2. Hierarchical Clustering

Hierarchical clustering was also performed to identify the groups of observations that were most similar to one another based on how they answered the questions. Patients within a cluster are similar to each other and different from ones in other clusters. We employed the approach of hierarchical clustering with Euclidean distance as measure of dissimilarity [26], and “ward.D2.” [27] as the algorithm. The Euclidean distance measure allows the algorithm to group observations that are closer together in terms of their characteristics, and “ward.D2” aims to minimize the total within-cluster variance. To determine the number of clusters to use, we used the “friedman” index, which decides the number of clusters based on the maximum difference between hierarchy levels [28]. This approach allowed us to identify the optimal number of clusters to use and create meaningful clusters based on the underlying structure of the data.

#### 2.2.3. Latent Class Analysis (LCA)

We defined the hypothesized unobserved classes through LCA. The process of LCA begins by fitting a baseline model with only one latent class. Then, the number of latent classes is increased iteratively, and the model is re-estimated at each step until an appropriate fit is obtained [29]. To assess the model’s fit and determine an appropriate number of latent classes, we use the statistical measures Bayesian information criterion (BIC) [30] and entropy [31]. BIC is a parsimony measure that balances model complexity with goodness of fit, while entropy measures the degree of uncertainty in assigning individuals to classes. An entropy value close to 1 indicates a high degree of certainty in class assignment, which is considered ideal.

## 3. Results

Of the 2380 participants used for analysis, the mean age was 53.1 years (SD 16.3), over half of the participants were female (59.0%), and about 50% of the participants were Black (Table 1).

### 3.1. Exploratory Factor Analysis

**Figure 1:**
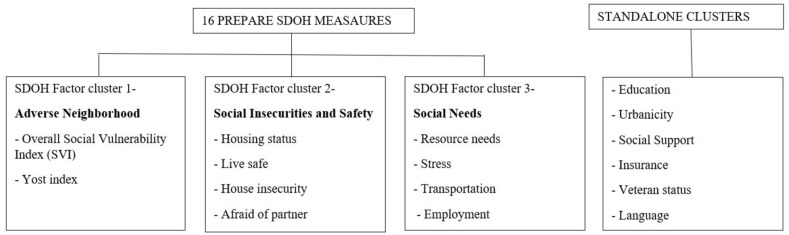
Structure of PRAPARE SDoH factors by factor analysis. Abbreviation: PRAPARE, Protocol for Responding to and Assessing Patient Assets, Risks, and Experiences.

We conducted an exploratory factor analysis on 16 Social Determinants of Health (SDoH) measures after excluding three variables (incarcerated, refugee, migrant) due to their low prevalence. The analysis yielded three clusters with eigenvalues greater than 1, indicating that they are meaningful clusters (Figure 1). The first factor found was labeled “Adverse Neighborhood”, based on the characteristics of two neighborhood level SDoH factors: overall Social Vulnerability Index (SVI) and Yost index. The second factor was labeled “Social insecurities and Safety” and consisted of four factors: housing status, live safe, house insecurity, and afraid of partner. The third factor was labeled “Social Needs”, which consisted of four factors: resource needs, stress, transportation, and employment (Figure 1). The root mean squared error of approximation was 0.02, which indicates that the exploratory factor analysis model fit was acceptable [32]. The results suggest that SDoH questions on the PRAPARE are not independent and can be grouped into meaningful factors.

### 3.2. Hierarchical Clustering

**Figure 2:**
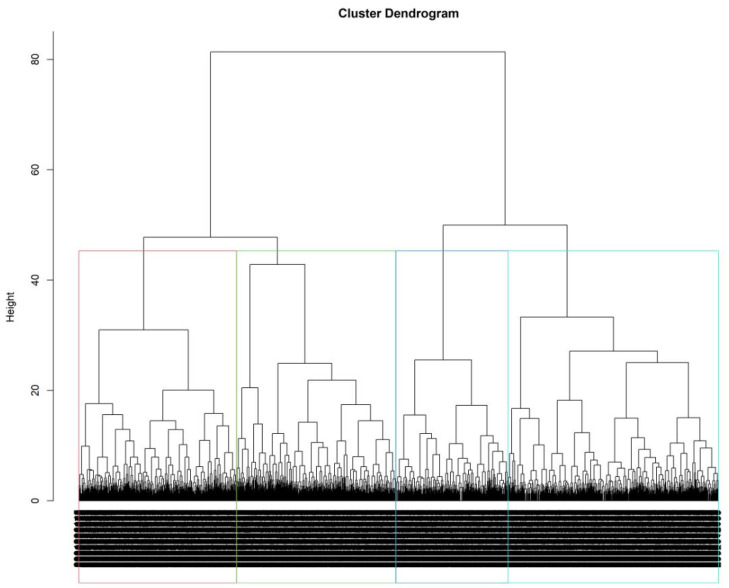
A dendrogram generated through the plotting of the hierarchical cluster object. Rectangles around the branches of a dendrogram highlight the corresponding clusters. Objects that are located at the same cluster share similar characteristics with each other.

We included all 22 variables (including age, sex, and gender) in the study, and classified age into three categories with a cutoff at 39 and 59. To determine the ideal number of clusters in the dataset, we utilized the “friedman” index, a statistical technique introduced in the Methods section, and explored cluster numbers ranging from 2 to 15. Based on the analysis, we identified four distinct clusters of patients. As shown in Table 2, we described the clusters according to SDoH and other demographics: (1) older, mostly optimal SDoH; (2) housing and resources needs, high stress, unemployed suboptimal SDoH; (3) predominantly male, most suboptimal SDoH reported; (4) predominantly middle aged, optimal SDoH. Table 2 presents the distribution of patients in each of these clusters, providing a summary of the findings. In Figure 2, we present a dendrogram that has been generated through the plotting of the hierarchical cluster object. This dendrogram provides a visual representation of the relationships and clustering patterns within our data. Each branch and grouping in the dendrogram signifies the degree of similarity or dissimilarity among the data points or entities being analyzed. Overall, this method shows different SDoH status and race-based clusters that exist among patients.

### 3.3. Latent Class Analysis

**Figure 3:**
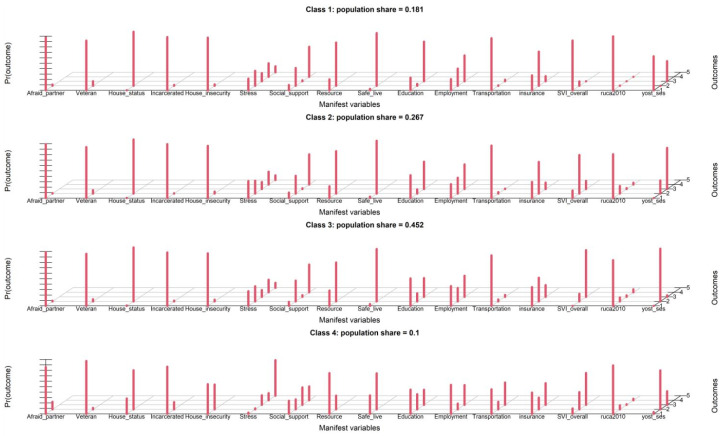
Estimation of the four-class basic latent class model; obtained by setting graphs = TRUE in the poLCA function call. Each group of red bars represents the conditional probabilities, by latent class, of being rated positively by each of the 16 SDoH measures. Taller bars correspond to conditional probabilities closer to 1 of a positive rating.

**Figure 4:**
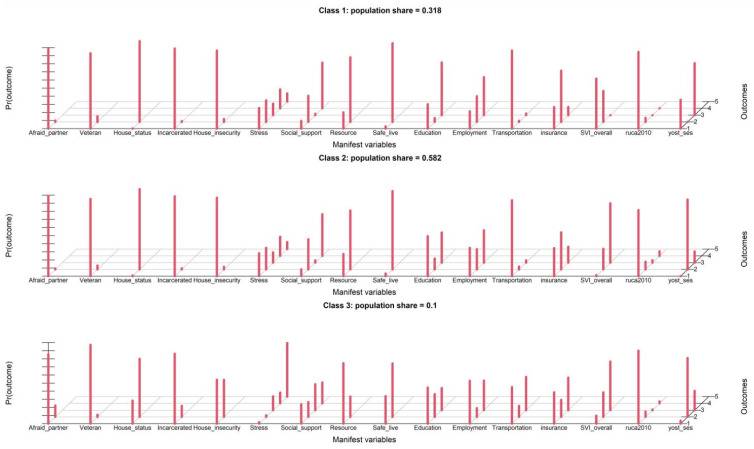
Estimation of the three-class basic latent class model; obtained by setting graphs = TRUE in the poLCA function call. Each group of red bars represents the conditional probabilities, by latent class, of being rated positively by each of the 16 SDoH measures. Taller bars correspond to conditional probabilities closer to 1 of a positive rating.

Gender, age, and race covariates were not included, and the latent class analysis identified four distinct classes of patients who shared similar response patterns based on the Bayesian Information Criterion (BIC), as shown in Table 3. The entropy of 0.745 indicated a high degree of certainty in class assignment, which suggests that the classes are distinct and well-defined. Our first look at the analysis revealed four classes that were labeled based on their socioeconomic status and social needs: (1). “High Socioeconomic Status, Low Social Needs”, (2). “Moderate Socioeconomic Status, Moderate Social Needs” (3). “Low Socioeconomic Status, High Social Needs” (4). “Very Low Socioeconomic Status, Very High Social Needs”. These four classes accounted for 18%, 27%, 45%, and 10% percentages of the population. Figure 3 in the article shows a screen capture of the estimation of the model, which provides a visual representation of the four estimated latent classes. After further inspection, we found that class 1 (Low Social Needs) and class 2 (Moderate Social Needs) were similar in nature. Moreover, there was minimal divergence in BIC between the model with three classes and the one with four classes (Table 3). To facilitate better interpretation and clinical usage, we opted for a three-class model, which resulted in an increased entropy of 0.841. These three classes represented 31.8%, 58.2%, and 10% of the population. The probabilities of being positively rated by each variable, based on latent class, are illustrated in Figure 4 for the three-class model. These classes, or SDoH phenotypes, were utilized in a clinical paper that linked phenotypes to health outcomes (under review). The classes were categorized as follows: (1) low-social risk; (2) adverse neighborhood SDoH; and (3) high-social risk.

## 4. Discussion

For this investigation, we conducted exploratory factor analysis, hierarchical clustering, and latent class analysis to uncover the patterns and relationships within Social Determinants of Health (SDoH) variables collected through the PRAPARE tool. These valuable statistical techniques can provide insights into the underlying structure of the data, identify key social determinants of health, and help to identify subgroups of patients who may require tailored interventions. Our results show how approaches can be utilized to achieve different objectives based on the needs of the researcher or study.

Due to the collinearity of SDoH factors, researchers have grappled with how to approach dealing with these measures, including clustering methods, creating indices using PCA, and mostly recently, polysocial risks scores [33]. The decision to use exploratory factor analysis, hierarchical clustering, and latent class analysis in our research reflects a deliberate effort to approach patient clustering from multiple angles, acknowledging the diverse aspects of the underlying data. Although these methods were initially applied in a patient setting, they hold the potential for broader applications in various settings, extending their value not only to healthcare, but also to diverse research endeavors.

Exploratory factor analysis allowed us to structure the multiple SDoH variables into three factors: adverse neighborhood, social insecurities and safety, and social needs. The confirmatory factor analysis supported the validity of this structure. This finding provides insights into the potential underlying structure of the data, and informing how future scales might be able to group concepts to describe specific domains, such as housing or food insecurity [34]. This result aligns with the study of Wan et al. [9], who identified three composite clusters among the 22 PRAPARE SDoH factors.

Simultaneously, hierarchical clustering provided a different lens, identifying four distinct clusters of patients: older, mostly optimal SDoH; housing and resources needs, high stress, unemployed suboptimal SDoH; predominantly male, most suboptimal SDoH reported; and predominantly middle aged, optimal SDoH, based on similarities in how they answered the questions. This approach offers a complementary perspective, focusing on patient groupings in a way that aligns with certain shared responses to specific questions [35]. To our knowledge, our study is the first to cluster PRAPARE patients using hierarchical clustering. Latent class analysis allowed us to identify three unobserved classes (low-social risk; adverse neighborhood SDoH; high-social risk) that influence the self-report or the measures of SDoH variables; it emphasized capturing patterns in responses that might not be immediately apparent through other clustering techniques. We ultimately used this approach to inform our outcome modeling of diabetes and obesity status in the original study.

While results varied by method, they did so only slightly, indicating the robustness of the patterns that we found in our particular data. The use of exploratory factor analysis, hierarchical clustering, and latent class analysis consistently revealed disparities in critical areas such as Social Vulnerability Index (SVI), housing status, living conditions, housing insecurity, and stress. For instance, hierarchical clustering identified significant differences in the percentage of low social vulnerability among clusters, with clusters 3 and 4 demonstrating lower rates than clusters 1 and 2. Similarly, in latent class analysis, class 2 (adverse neighborhood SDoH) and class 3 (high social risk) contributed to a much higher rate of high social vulnerability compared to class 1 (low social risk). On the other hand, each technique offered unique advantages and perspectives, contributing to a well-rounded interpretation of the complex relationships within the dataset. Considering this, investigators need to determine what their objective is with the data when selecting a method—(1) to identify underlying constructs of questions asked to individuals (factor analysis); or (2) to identify segments or clusters of people based on shared characteristics (hierarchical clustering or LCA). Here we wanted to examine the latter, which could help healthcare providers develop more targeted and effective strategies for addressing health disparities and promoting health equity. On the other hand, factor analysis is helpful when developing screeners and questions pertinent to SDoH, and in discovering domains that may exist in the data or among patients.

Our study has several implications from both a research and clinical perspective. For instance, FA may prove to be more useful when developing social risk and/or SDoH screeners with good content validity. Hierarchical clustering may help to pool patients based solely on shared responses to inform clinical approaches and interventions. Latent class analysis may help to identify subtypes of patients based on shared SDoH for intervention development and tailoring. Investigators should choose approaches based on their intended outcomes; however, it is important to highlight here that all three approaches produced similar results and confirm that SDoH does indeed cluster into distinct categories.

Our study has several limitations to mention. One limitation is that the applied techniques are reliant on the quality and completeness of the data gathered with the PRAPARE tool. If the data are incomplete or inaccurate, it can affect the validity and reliability of the results. Therefore, it is important to ensure that the data are collected using standardized and validated measures, and that any missing data are handled appropriately. While our results here are a demonstration of different methods, it is important to highlight this limitation. It is also important to consider the potential ethical implications of using these techniques, which was not the main focus of this study. For example, clustering or latent class analysis could potentially result in stigmatization or discrimination against certain subgroups of patients. Therefore, it is important to ensure that the results are used in a responsible and ethical manner, and that appropriate safeguards are put in place to protect patient privacy and confidentiality.

## 5. Conclusions

In our study, which involved 2380 participants and examined 19 Social Determinants of Health (SDoH) variables, three factors emerged through factor analysis: adverse neighborhood, social insecurities and safety, and social needs. Hierarchical clustering revealed four distinct patient clusters: older, mostly optimal SDoH; housing and resource needs, high stress, unemployed suboptimal SDoH; predominantly male, most suboptimal SDoH reported; and predominantly middle-aged, optimal SDoH. Furthermore, latent class analysis identified three unobserved classes related to social risk: low-social risk, adverse neighborhood SDoH, and high-social risk. These results demonstrate the reliability and validity of scoring tools. Future work should explore the use of these tools for improving population health outcomes.

Our paper primarily targets healthcare professionals who utilize the PRAPARE tool in real-world settings, and healthcare researchers interested in disseminating descriptive findings on SDoH and assessing the relationships between SDoH and patient outcomes. While this paper involves statistical methodologies, it is crafted to be accessible to a broad audience, including practitioners and individuals with varying levels of statistical proficiency.

## Figures and Tables

**Table 1:** Characteristics of the study population.

	Total (N = 2380)	Coded
Race		
White	1182 (49.7%)	1
Black or Other	1198 (50.3%)	2
Gender		
Male	975 (41.0%)	1
Female	1405 (59.0%)	2
Age in year		
Mean (SD)	53.1 (16.3)	
Afraid of partner		
No	2318 (97.4%)	1
Yes	62 (2.6%)	2
Migrant		
No	2366 (99.4%)	1
Yes	14 (0.6%)	2
Refugee		
No	2371 (99.6%)	1
Yes	9 (0.4%)	2
Veteran		
No	2255 (94.7%)	1
Yes	125 (5.3%)	2
House status		
I have housing	2299 (96.6%)	1
I do not have housing	81 (3.4%)	2
Incarcerated		
No	2320 (97.5%)	1
Yes	60 (2.5%)	2
House insecurity		
Not Worried About Losing Housing	2201 (92.5%)	1
Worried About Losing Housing	179 (7.5%)	2
Stress		
Not at all	574 (24.1%)	1
A little bit	589 (24.7%)	2
Quite a bit	324 (13.6%)	3
Somewhat	537 (22.6%)	4
Very much	356 (15.0%)	5
Social support		
More than 5 times a week	1202 (50.5%)	1
3 to 5 times a week	804 (33.8%)	2
1 or 2 times a week	246 (10.3%)	3
Less than once a week	128 (5.4%)	4
Resource		
None reported	1670 (70.2%)	1
Needed	710 (29.8%)	2
Language		
English	2329 (97.9%)	1
Other	51 (2.1%)	2
Safe live		
Yes	2231 (93.7%)	1
No	149 (6.3%)	2
Education		
More than high school	1073 (45.1%)	1
High school diploma or GED	1018 (42.8%)	2
Less than a high school degree	289 (12.1%)	3
Employment		
Employed	612 (25.7%)	1
Retired	997 (41.9%)	2
Unemployed	771 (32.4%)	3
Transportation		
No need	2135 (89.7%)	1
Non-medical need	154 (6.5%)	2
Medical need	91 (3.8%)	3
Insurance		
Private	1162 (48.8%)	1
Public	774 (32.5%)	2
Self-pay	444 (18.7%)	3
Overall SVI		
Low	508 (21.3%)	1
Moderate	714 (30.0%)	2
High	1158 (48.7%)	3
Urbanicity		
Metropolitan	2060 (86.6%)	1
Micropolitan	188 (7.9%)	2
Small Town	88 (3.7%)	3
Rural	44 (1.8%)	4
Yost Index		
High	279 (11.7%)	1
Moderate	734 (30.8%)	2
Low	1367 (57.4%)	3

**Table 2:** Characteristics of the study population in each cluster produced using hierarchical clustering.

	Total (N = 2380)	Cluster 1 (N = 783)	Cluster 2 (N = 587)	Cluster 3 (N = 593)	Cluster 4 (N = 417)
Cluster Description		Older, mostly optimal SDoH	Housing and resources need, high stress, unemployed suboptimal SDoH	Predominantly male, most suboptimal SDoH reported	Predominantly middle aged, optimal SDoH
Race					
White	1182 (49.7%)	398 (50.8%)	287 (48.9%)	335 (56.5%)	162 (38.8%)
Black or Other	1198 (50.3%)	385 (49.2%)	300 (51.1%)	258 (43.5%)	255 (61.2%)
Afraid of partner					
No	2318 (97.4%)	775 (99.0%)	564 (96.1%)	567 (95.6%)	412 (98.8%)
Yes	62 (2.6%)	8 (1.0%)	23 (3.9%)	26 (4.4%)	5 (1.2%)
Migrant					
No	2366 (99.4%)	778 (99.4%)	584 (99.5%)	588 (99.2%)	416 (99.8%)
Yes	14 (0.6%)	5 (0.6%)	3 (0.5%)	5 (0.8%)	1 (0.2%)
Refugee					
No	2371 (99.6%)	780 (99.6%)	583 (99.3%)	593 (100%)	415 (99.5%)
Yes	9 (0.4%)	3 (0.4%)	4 (0.7%)	0 (0%)	2 (0.5%)
Veteran					
No	2255 (94.7%)	729 (93.1%)	560 (95.4%)	565 (95.3%)	401 (96.2%)
Yes	125 (5.3%)	54 (6.9%)	27 (4.6%)	28 (4.7%)	16 (3.8%)
House status					
I have housing	2299 (96.6%)	773 (98.7%)	568 (96.8%)	547 (92.2%)	411 (98.6%)
I do not have housing	81 (3.4%)	10 (1.3%)	19 (3.2%)	46 (7.8%)	6 (1.4%)
Incarcerated					
No	2320 (97.5%)	773 (98.7%)	574 (97.8%)	562 (94.8%)	411 (98.6%)
Yes	60 (2.5%)	10 (1.3%)	13 (2.2%)	31 (5.2%)	6 (1.4%)
House insecurity					
Not Worried About Losing Housing	2201 (92.5%)	761 (97.2%)	534 (91.0%)	502 (84.7%)	404 (96.9%)
Worried About Losing Housing	179 (7.5%)	22 (2.8%)	53 (9.0%)	91 (15.3%)	13 (3.1%)
Gender					
Male	975 (41.0%)	302 (38.6%)	227 (38.7%)	281 (47.4%)	165 (39.6%)
Female	1405 (59.0%)	481 (61.4%)	360 (61.3%)	312 (52.6%)	252 (60.4%)
Stress					
Not at all	574 (24.1%)	373 (47.6%)	37 (6.3%)	11 (1.9%)	153 (36.7%)
A little bit	589 (24.7%)	306 (39.1%)	0 (0%)	36 (6.1%)	247 (59.2%)
Quite a bit	324 (13.6%)	75 (9.6%)	150 (25.6%)	84 (14.2%)	15 (3.6%)
Somewhat	537 (22.6%)	26 (3.3%)	270 (46.0%)	239 (40.3%)	2 (0.5%)
Very much	356 (15.0%)	3 (0.4%)	130 (22.1%)	223 (37.6%)	0 (0%)
Social support					
More than 5 times a week	1202 (50.5%)	742 (94.8%)	0 (0%)	459 (77.4%)	1 (0.2%)
3 to 5 times a week	804 (33.8%)	13 (1.7%)	417 (71.0%)	46 (7.8%)	328 (78.7%)
1 or 2 times a week	246 (10.3%)	4 (0.5%)	150 (25.6%)	16 (2.7%)	76 (18.2%)
Less than once a week	128 (5.4%)	24 (3.1%)	20 (3.4%)	72 (12.1%)	12 (2.9%)
Resource					
None reported	1670 (70.2%)	634 (81.0%)	330 (56.2%)	368 (62.1%)	338 (81.1%)
Needed	710 (29.8%)	149 (19.0%)	257 (43.8%)	225 (37.9%)	79 (18.9%)
Language					
English	2329 (97.9%)	765 (97.7%)	578 (98.5%)	579 (97.6%)	407 (97.6%)
Other	51 (2.1%)	18 (2.3%)	9 (1.5%)	14 (2.4%)	10 (2.4%)
Safe live					
Yes	2231 (93.7%)	757 (96.7%)	543 (92.5%)	530 (89.4%)	401 (96.2%)
No	149 (6.3%)	26 (3.3%)	44 (7.5%)	63 (10.6%)	16 (3.8%)
Education					
More than high school	1073 (45.1%)	397 (50.7%)	234 (39.9%)	268 (45.2%)	174 (41.7%)
High school diploma or GED	1018 (42.8%)	296 (37.8%)	293 (49.9%)	229 (38.6%)	200 (48.0%)
Less than a high school degree	289 (12.1%)	90 (11.5%)	60 (10.2%)	96 (16.2%)	43 (10.3%)
Employment					
Employed	612 (25.7%)	219 (28.0%)	119 (20.3%)	148 (25.0%)	126 (30.2%)
Retired	997 (41.9%)	359 (45.8%)	235 (40.0%)	248 (41.8%)	155 (37.2%)
Unemployed	771 (32.4%)	205 (26.2%)	233 (39.7%)	197 (33.2%)	136 (32.6%)
Transportation					
No need	2135 (89.7%)	744 (95.0%)	517 (88.1%)	479 (80.8%)	395 (94.7%)
Non-medical need	154 (6.5%)	20 (2.6%)	37 (6.3%)	83 (14.0%)	14 (3.4%)
Medical need	91 (3.8%)	19 (2.4%)	33 (5.6%)	31 (5.2%)	8 (1.9%)
AGE					
<=39	537 (22.6%)	158 (20.2%)	133 (22.7%)	167 (28.2%)	79 (18.9%)
~<=59	973 (40.9%)	291 (37.2%)	245 (41.7%)	241 (40.6%)	196 (47.0%)
>=60	870 (36.6%)	334 (42.7%)	209 (35.6%)	185 (31.2%)	142 (34.1%)
Insurance					
Private	1162 (48.8%)	414 (52.9%)	301 (51.3%)	250 (42.2%)	197 (47.2%)
Public	774 (32.5%)	247 (31.5%)	179 (30.5%)	205 (34.6%)	143 (34.3%)
Self-pay	444 (18.7%)	122 (15.6%)	107 (18.2%)	138 (23.3%)	77 (18.5%)
Overall SVI					
Low	508 (21.3%)	225 (28.7%)	139 (23.7%)	84 (14.2%)	60 (14.4%)
Moderate	714 (30.0%)	236 (30.1%)	181 (30.8%)	181 (30.5%)	116 (27.8%)
High	1158 (48.7%)	322 (41.1%)	267 (45.5%)	328 (55.3%)	241 (57.8%)
Urbanicity					
Metropolitan	2060 (86.6%)	719 (91.8%)	522 (88.9%)	438 (73.9%)	381 (91.4%)
Micropolitan	188 (7.9%)	62 (7.9%)	58 (9.9%)	38 (6.4%)	30 (7.2%)
Small Town	88 (3.7%)	0 (0%)	2 (0.3%)	86 (14.5%)	0 (0%)
Rural	44 (1.8%)	2 (0.3%)	5 (0.9%)	31 (5.2%)	6 (1.4%)
Yost index					
High	279 (11.7%)	153 (19.5%)	78 (13.3%)	21 (3.5%)	27 (6.5%)
Moderate	734 (30.8%)	241 (30.8%)	188 (32.0%)	180 (30.4%)	125 (30.0%)
Low	1367 (57.4%)	389 (49.7%)	321 (54.7%)	392 (66.1%)	265 (63.5%)

**Table 3:** Fit statistics of different latent classes using the latent class analysis method.

Model	BIC	aBIC	cAIC	Likelihood-Ratio	Entropy	Smallest Class Size
Model 1	49,573.21	49,481.07	49,602.21	14,692.14	--	(%)
Model 2	47,852.33	47,664.87	47,911.33	12,738.02	0.879	34
**Model 3**	**46,913.15**	46,630.38	**47,002.15**	11,565.59	**0.841**	10
Model 4	**46,822.80**	46,444.72	**46,941.80**	11,242.00	**0.745**	10
Model 5	46,834.79	46,361.38	46,983.79	11,020.74	0.715	7
Model 6	46,870.66	46,301.93	47,049.66	10,823.36	0.689	6

Note: bolded values indicate the criteria used to compare Model 3 and 4 to determine the best model fit.

## Data Availability

The data presented in this study are available on request from the corresponding author due to privacy and ethical restriction.
